# Repeated PTZ Treatment at 25-Day Intervals Leads to a Highly Efficient Accumulation of Doublecortin in the Dorsal Hippocampus of Rats

**DOI:** 10.1371/journal.pone.0039302

**Published:** 2012-06-29

**Authors:** Ana-Maria Buga, Raluca Vintilescu, Adrian Tudor Balseanu, Oltin Tiberiu Pop, Costin Streba, Emil Toescu, Aurel Popa-Wagner

**Affiliations:** 1 Department of Psychiatry, Molecular Psychiatry, Rostock University Medical School, Rostock, Germany; 2 Department of Functional Sciences, University of Medicine and Pharmacy, Craiova, Romania; 3 Department of Clinical and Experimental Medicine, College of Medical and Dental Sciences University of Birmingham Edgbaston, Birmingham, United Kingdom; Children‘s Hospital of Philadelphia, United States of America

## Abstract

**Background:**

Neurogenesis persists throughout life in the adult mammalian brain. Because neurogenesis can only be assessed in postmortem tissue, its functional significance remains undetermined, and identifying an *in vivo* correlate of neurogenesis has become an important goal. By studying pentylenetetrazole-induced brain stimulation in a rat model of kindling we accidentally discovered that 25±1 days periodic stimulation of Sprague-Dawley rats led to a highly efficient increase in seizure susceptibility.

**Methodology/Principal Findings:**

By EEG, RT-PCR, western blotting and immunohistochemistry, we show that repeated convulsive seizures with a periodicity of 25±1 days led to an enrichment of newly generated neurons, that were BrdU-positive in the dentate gyrus at day 25±1 post-seizure. At the same time, there was a massive increase in the number of neurons expressing the migratory marker, doublecortin, at the boundary between the granule cell layer and the polymorphic layer in the dorsal hippocampus. Some of these migrating neurons were also positive for NeuN, a marker for adult neurons.

**Conclusion/Significance:**

Our results suggest that the increased susceptibility to seizure at day 25±1 post-treatment is coincident with a critical time required for newborn neurons to differentiate and integrate into the existing hippocampal network, and outlines the importance of the dorsal hippocampus for seizure-related neurogenesis. This model can be used as an in vivo correlate of neurogenesis to study basic questions related to neurogenesis and to the neurogenic mechanisms that contribute to the development of epilepsy.

## Introduction

Pentylenetetrazole (PTZ) is a GABA-A antagonist. By studying PTZ-induced brain stimulation in a rat model of aging we discovered that periodic PTZ-stimulation of Sprague-Dawley rats at 25±1 day intervals led to a highly efficient increase in seizure susceptibility [1]. Specifically, a high proportion (over 70%) of animals reached full kindling status after only two subconvulsant doses of pentylenetetrazole (PTZ) (30 mg/kg) given 25 days apart; we dubbed this model the *critical time window kindling model* [1]. Although this work suggested that the persistent expression of several classes of brain plasticity-associated proteins may be required for the maintenance of kindling status, the results do not explain the 25±1 day periodicity of seizure susceptibility. Hence, it seemed likely that other cellular events might be involved. Subsequent studies on stimulation-induced neurogenesis in the adult hippocampus have revealed that, at 3 wks after stimulation, the newly generated neurons have an intrinsic excitability [2]–[4]. Therefore, in the present study, we tested the hypothesis that the critical interval determining the exquisite sensitivity of the brain to repeated exposure to PTZ is governed by the *timing of neurogenesis* in affected regions.

## Materials and Methods

All experimental procedures were approved by the Institutional Animal Care and Use Committee of the State M-V (approval number: LALLF M-V/TSD/7221.3-1.1-044/08) and by the Institutional Animal Care and Use Committee of the Medical University of Craiova.

### Animals

Three-mo-old male Sprague-Dawley rats were maintained on a 12 h light/dark cycle and allowed free access to food and water. The body weights ranged from 320–400 g. A total of 212 rats were used.

### Administration of pentylenetetrazole (PTZ)

Each experiment was performed with groups of 15–20 young-adult male rats for each timepoint at which the animals were sacrificed.

#### Experiment 1: A single seizure episode and cytogenesis

**Figure 1:**
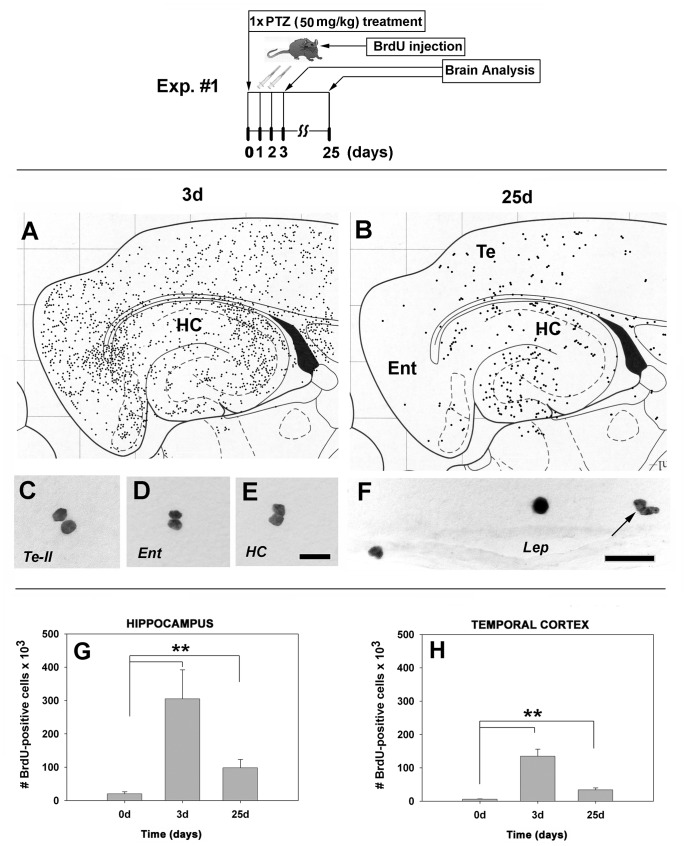
Diagrams. (*Upper panel*): Flow diagram of Exp. #1 regarding times and type of treatment with pentylenetetrazole. *(Lower panel):* Schematic diagram of the distribution of proliferative (BrdU-positive) cells in the brain at day 25 after a single administration of a convulsive dose of PTZ. Note the change from a widespread distribution (**A**) to a more restricted distribution at 25 days post-seizure (**B**). In control rats, there were only a few BrdU-positive cells, often in a duplex, mitosis-like state (**C, D, E**). Many proliferative cells in PTZ-treated animals appeared to enter the brain from the circulation via leptomeningeal blood vessels (**F**, arrow points to a mitosis-like state). (**G**, **H**): Quantitation of BrdU-positive cells. One episode of convulsive seizure causes, at day 3, dramatically increased BrdU-positive cell numbers in the hippocampus (15-fold over controls; p = 0.001) (**G**) and temporal neocortex (22.5-fold over controls; p = 0.001) (**H**). Although the numbers of BrdU-positive cells decreased dramatically by day 25, their number remained, nonetheless, at relatively high levels in the hippocampus (4.8-fold; p = 0.001)(**G**) and temporal neocortex (5.6-fold; p = 0.001) (**H**) over control levels. N = 15 rats for each timepoint. *Abbreviations*: *Te-II*, temporal neocortex layer II; *Ent*, entorhinal neocortex; *HC*, hippocampus; *lep*, leptomeninx. Bars: (**C, D, E**), 20 µm; (**F**), 30 µm.

Convulsive seizures were elicited by a single convulsant injection of PTZ (50 mg/kg). To label the newly generated cells, rats were given injections of bromodeoxyuridine (BrdU; 50 mg/kg body weight, i.p.; Roche, Germany) at days 1–3 post-seizure (Fig. 1). The animals were sacrificed at day 24 post-seizure and the distribution of proliferating, BrdU-positive cells in the brain analyzed as described below.

#### Experiment 2: Full kindling status and cytogenesis

**Figure 2:**
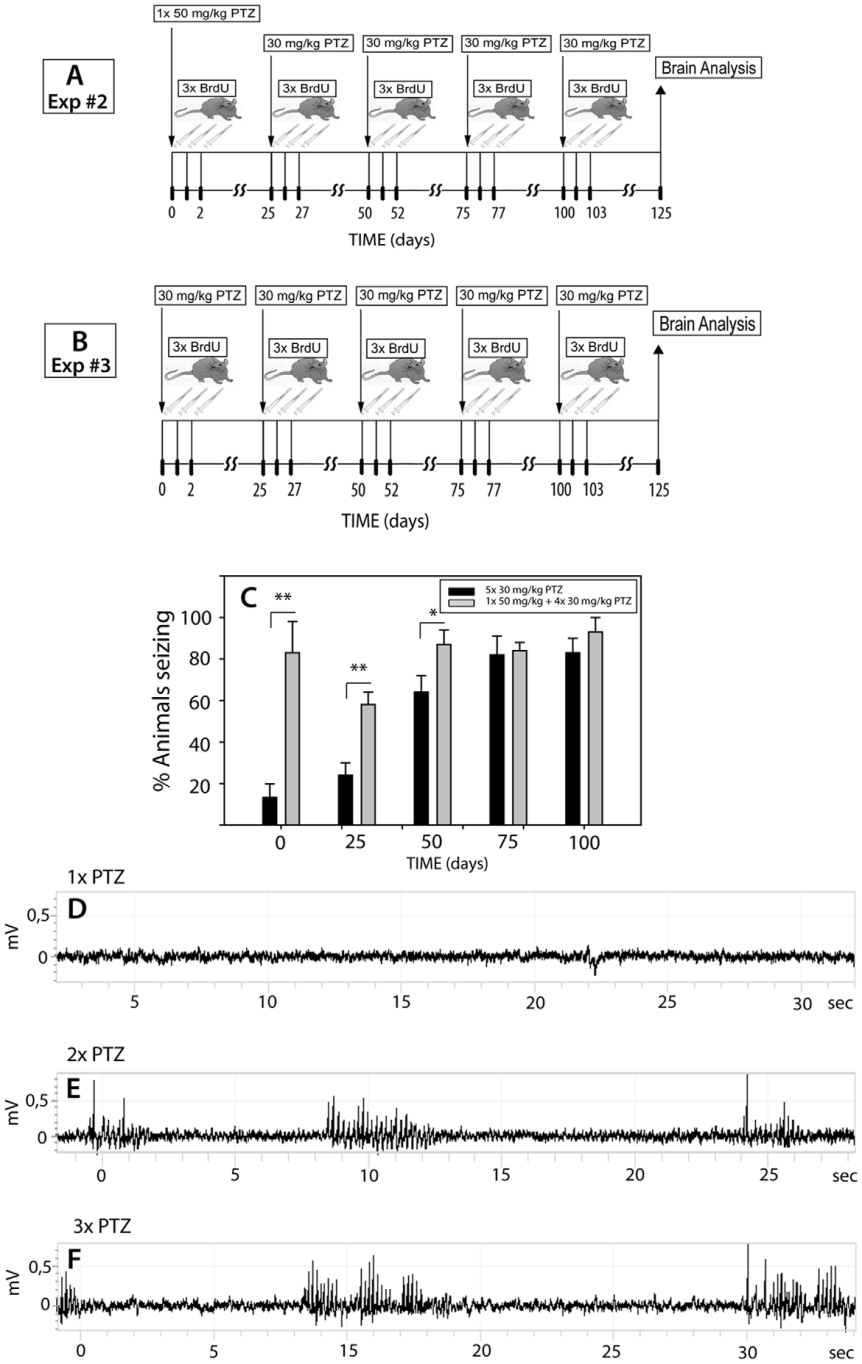
Flow diagrams of the experimental design regarding times and type of treatment and EEG recording. After Exp. #2 (**A**) the proportion of rats that achieved full kindling status reached 80% (**C**; p = 0.001). By comparison, 31% of rats administered a subconvulsive dose of PTZ at all times (Exp. #3)(**B**) reached full kindling status after two injections and up to 81% of animals reached full kindling status after the fourth treatment (**C;** p = 0.001). (**D**–**F**): A subconvulsive PTZ treatment elicited intermittent non-ictal events that are typically dependent on the behavioral state of the animal (active or passive wakefulness)(**D**). After the second PTZ injection, the animals usually demonstrated mild multifocal body jerks (**E**). After the third PTZ injection, the animals showed typical seizure activity associated with motor arrest (**F**).

To induce full kindling status (scores 4 and 5 according to Racine) [5], rats were primed first with a convulsive dose of PTZ at 50 mg/kg. Kindling criterion was maintained by four subsequent subconvulsive doses of PTZ (30 mg/kg) given 25±1 days apart [1]. In order to evaluate cell accumulation and cellular phenotype, rats were given BrdU (50 mg/kg, i.p.) at days 1, 2 and 3 after each PTZ treatment (Fig. 2A). The animals were sacrificed at day 125 post-seizure and the brains analyzed as described below.

#### Experiment 3: Achievement of kindling status by repeated treatment with a subconvulsive dose of PTZ

To induce full kindling status, rats were treated i.p. with subconvulsant doses of PTZ at a concentration of 30 mg/kg, given 25 days apart over a total period of 100 days [1]. In order to evaluate cell accumulation and cellular phenotype, rats were given BrdU (50 mg/kg, i.p.) at days 1, 2 and 3 after each PTZ treatment (Fig. 2B). The animals were sacrificed at day 125 post-seizure and the brains analyzed as described below.

#### Experiment 4: Neurogenesis stimulation by treatment with L-NAME

Treatment with the Nω-nitro-L-arginine methyl ester nitric oxide synthase inhibitors such as Nω-nitro-L-arginine methyl ester (L-NAME) has been shown to stimulate neurogenesis in the rat hippocampus [6], [7]. To test the neurogenesis hypothesis of kindling more directly, rats were treated i.p. with a subconvulsant dose of PTZ at a concentration of 30 mg/kg on day 0. During the PTZ treatment we observed a transient increase in blood pressure that peaked at 6 hrs post-administration (unpublished). In order to avoid the additional effect of increased blood pressure caused by L-NAME [6], [7], we started the L-NAME treatment (90 mg/kg per day, i.p.) on day 1 and completed treatment on day 24.

Initially, L-NAME was administered for 14 days following published protocols [6]. However, the effect this duration of L-NAME treatment on seizure susceptibility was not significantly different between control and treated animals. For this reason, the L-NAME treatment was extended to 24 days. A second PTZ injection was given on day 25 and the brains were analyzed on day 50 post-seizure. Control rats for all groups received i.p. physiological saline.

### EEG Recordings

Under anesthesia, animals were positioned in a stereotaxic frame, a midline incision was made along the scalp, which was reflected bilaterally and 2 burr holes were made in the skull (bregma for positive wire and lambda for negative wire), but did not perforate the dura. The electrode wire was secured to the skull surface with dental acrylic. The skin around the headset was sutured and the animals were allowed to recover from anesthesia. Lesioned animals recovered for 1 week before monitoring, to ensured complete recovery from anesthesia and the local effects of surgery. Qualitative EEG analysis was performed by visual examination of the record with attention paid to generalized and intermittent focal epileptic activities. Epileptic activities were distinguished from non-epileptic activities based on changes in waveform morphology, amplitude, and frequency, as well as the associated absence or presence of behavioural change.

### Tissue Processing

Following various survival times, the rats were deeply anesthetized with 3% halothane in 75% nitrous oxide/25% oxygen, and perfused with buffered saline. The brains were removed and bisected midsagitally. One half of each brain (left and right hemispheres were collected alternately) was fixed in 4% paraformaldehyde dissolved in 50 mM phosphate buffer (pH 7.2) for 24 hr, cryoprotected in 20% sucrose in 10 mM phosphate-buffered saline (pH 7.2), and stored at −70°C. This hemisphere was used for immunohistochemistry; the hippocampus and temporal neocortex from the contralateral hemisphere were used for biochemical analyses.

### RNA Isolation

Total RNA was isolated using TRIzol reagent (Invitrogen Life Technologies, Karlsruhe, Germany), as described by the manufacturer, followed by DNase 1 (Invitrogen Life Technologies, Karlsruhe, Germany) digestion and further purification using the RNeasy Mini extraction kit (Qiagen, Hilden, Germany). Purified total RNA was used for real-time PCR quantification.

### Real-time Reverse Transcriptase PCR (RT-PCR)

Total RNA was isolated from individual frozen hippocampal homogenates using the RNeasy Mini extraction kit (Qiagen, Hilden, Germany). For RT-PCR, 200 ng of total RNA was reverse-transcribed using random hexamers and the reverse transcription reagents (Superarray, Frederick, MD, USA). The assay was set up with 8 ng of reverse-transcribed RNA and GAPDH as an internal normalizer. Intron-spanning primer specific for DCX and nestin were supplied by BIOMOL (Hamburg, Germany). Amplification reactions were performed using a kit supplied by BioRad on a real-time PCR cycler (MyiQ, Thermal Cycler, BioRad, Munich).

### Immunohistochemistry

Sections (25 µm-thick) were cut on a freezing microtome and processed for immunohistochemistry as free-floating material. To obtain uniform staining of all tissue sections, we used an automated device (PCT, Device assembly for preparing and analyzing tissue for microscopic examinations; patents: DE 199 45 621 A1 and WO 01/22052 A1, 2001) that makes it possible to process and stain all sections simultaneously under identical conditions. Using the automated staining device, immunohistochemistry was conducted as previously described [1]. Briefly, after incubation with blocking solutions containing 3% donkey serum/10 mmol/l PBS/0.3% Tween 20, tissue sections were exposed overnight at 4°C to one of the following antibodies: (1) guinea pig anti-doublecortin (DCX) (1∶8000, Millipore, Schwalbach, Germany); (2) mouse anti-nestin (1∶3000, Millipore, Schwalbach, Germany); or (3) mouse anti NeuN (1∶1000, Millipore, Schwalbach, Germany), each diluted in PBS containing 3% normal donkey serum and 0.3% Tween 20. After extensive washing in PBS containing 0.3% Tween, sections were incubated overnight at 4°C with host-specific biotinylated seconday antibodies (Dianova, Hamburg, Germany) diluted 1∶2000 in PBS containing 1% normal donkey serum and 0.3% Tween 20. Finally sections were incubated with ABC Elite reagent (Vectastain Elite Kit, Linaris, Mannheim, Germany) diluted 1∶100 in PBS containing 0.3% Tween 20. The antibody complex was then visualized manually with 0.025% 3′,3′ diaminobenzidine (DAB) and 0.005% hydrogen peroxide in 100 mmol/l Tris buffer (pH 7.5).

For BrdU detection, free-floating sections were pre-treated with 50% formamide, 0.3 M NaCl, 10 mM sodium citrate at 65°C for 2 h, incubated in 2 M HCl at 37°C for 45 min, and rinsed in 0.1 M borate buffer (pH 8.5) at room temperature for 10 min. After neutralization, sections were incubated in blocking solution containing 10% lamb serum, 0.3% Triton X-100 in PBS overnight at 4°C, followed by mouse anti-BrdU antibody (1∶300, Roche, Mannheim, Germany) at 4°C for 24 hr. Sections were washed with PBS, incubated with biotinylated donkey anti-rat secondary antibody (1∶2000; Dianova, Hamburg, Germany) followed by the avidin–biotin peroxidase complex (Linaris, Mannheim, Germany) for 24 hrs. The horseradish peroxidase reaction was detected with 0.05% DAB and 0.03% H_2_O_2_. To visualize nuclei, some sections were counterstained with methyl green.

### Cell Counting

A quantitative estimate of the number of BrdU-, and DCX-immunopositive cells was obtained by counting cells on every tenth section across the entire hippocampal volume. To this end, a sequence of confocal counting images of 161×242×25 µm, spaced 0.1 µm apart across a 25 µm-thick section and covering 30% of the infarcted area, was taken for fluorescently labeled cells as previously described [8]. The resulting images were loaded into the 3-D analysis software “Volocity” (IMPROVISION, Coventry, UK) and computed using a Macintosh computer. The relative mean number of BrdU-positive cells was then calculated per group and time point and treatment by multiplying the number of cells per section times 3.3 (the counting boxes that were quantitated covered one third of the area of each section) times the section interval of 10.

Because of the asymmetry in the distribution of DCX, however, for DCX we counted every 5^th^ section in the dorsal hippocampus and every 10^th^ section in the ventral hippocampus.

### Cellular Phenotyping

#### Neuronal phenotype

Sections were double-immunolabeled with guinea pig anti-DCX antibodies (1∶4000, Millipore, Schwalbach, Germany) and rat anti-BrdU antibodies (1∶1000; AdSerotec, Duesseldorf, Germany). The antigen-antibody complexes were visualized with donkey anti-guinea pig Cy2-conjugated antibodies (1∶2000) and donkey anti-rat or donkey anti-mouse Rhodamine-conjugated antibodies (1∶3000), respectively. In other experiments, sections were double-immunolabeled with mouse anti-NeuN antibodies (1∶1000, Millipore, Schwalbach, Germany) and rat anti-BrdU antibodies (1∶2000; AdSerotec, Duesseldorf, Germany). The antigen-antibody complexes were detected with donkey anti-mouse Cy2-conjugated antibodies (1∶2000) and donkey anti-rat rhodamine-conjugated antibodies (1∶3000; Dianova, Hamburg, Germany), respectively.

### Western Blotting

For immunoblotting, SDS-PAGE-separated proteins were transferred to nitrocellulose membranes by semidry blotting. The membrane was then incubated with guinea pig anti-DCX antibodies (1∶4000, Millipore, Schwalbach, Germany) and the antigen-antibody complex was detected with goat anti-guinea pig-conjugated alkaline phosphatase antibodies (1∶8000; Jackson ImmunoResearch, West Grove, PA) using Luminol (Pierce, Amsterdam, Holland) as a substrate. For normalization, the immunoblots were incubated with rabbit anti-GAPDH antibodies (1∶5000, clone 14C10, Cell Signaling Technology, Danvers, MA). The immunoblots were scanned and analyzed with Kodak 1D Software.

### Microscopy

For light microscopy, a Nikon Eclipse microscope (Duesseldorf, Germany) was used. The digital images were arranged and labeled using Adobe Photoshop. Confocal analysis of sections was performed using a Nikon Eclipse microscope equipped with a laser device from Visitech (Munich, Germany). 3-dimensional reconstruction of overlapping antigens was achieved by taking a sequence of confocal images that were spaced 0.1 µm apart across a 25 µm-thick section. The resulting images were loaded into the 3-D analysis software “Volocity” (IMPROVISION, Coventry, UK) and computed using a MacIntosh computer.

### Statistical Analysis

Data are presented as means ± SD of two to five independent experiments. All data, including behavioural scoring, EEG parameters or cell counting were scored and measured in a reviewer-blind manner. The results of cell quantitation were evaluated by one-way ANOVA followed by Tukey post-hoc analyses using SPSS software (SPSS Inc., Chicago, Illinois). The level of significance was set at p< = 0.05, two-tailed test. Physiological data were analyzed by one-way repeated measures analysis of variance on ranks.

## Results

### Experiment 1: Seizure is Associated with Selective Cytogenesis in Brain

A single episode of PTZ-induced convulsive seizure caused the appearance at day 3 of large numbers of BrdU-positive cells having a non-preferential distribution in the entorhinal and temporal neocortices as well as the hippocampal formation (Fig. 1A). Many of these cells were in a mitosis-like state in the dentate gyrus, polymorphic cell layer of the hippocampus, and neocortex (not shown). After 25 days, most of the cells died but, unexpectedly, there was also a selective survival of BrdU-positive cells, especially those located in the hippocampus and temporal neocortex (Fig. 1B).

In control rats, there were only a few BrdU-immunopositive cells that were scattered randomly, often in a doubled, mitosis-like state, especially in the temporal neocortex (Fig.1 C), entorhinal neocortex (Fig.1 D) and the hippocampus (Fig. 1E). Many proliferative cells in PTZ-treated animals appeared to have entered the brain via the leptomeninges; many of these cells retained a mitosis-like appearance (Fig. 1F, arrow).

By day 3 post-seizure, the number of BrdU-positive cells (BrdU+) increased 16-fold in the hippocampus (Fig. 1G) and 10-fold in the temporal neocortex (Fig. 1H) of PTZ-treated rats. After 25 days, the number of BrdU+ cells remained high in the hippocampus and the temporal neocortex, albeit at much lower levels than at day 3 (Fig. 1G, H).

### Experiment 2: Full Kindling Status is Achieved after 3 Doses of PTZ

In this experiment, the rats were primed with a convulsive dose (50 mg/kg) of PTZ followed by four subconvulsive doses (30 mg/kg) of PTZ given 25 days apart, as shown in Fig. 2A. After two injections of a subconvulsive dose of PTZ, up to 86% of rats achieved full kindling status. Thereafter, the proportion of animals showing full kindling status remained essentially constant (Fig. 2C). By comparison, 31% of rats administered a subconvulsive dose of PTZ at all times reached full kindling status after two injections (Fig. 2C), and up to 81% reached full kindling status after the fourth treatment (Fig. 2C). In contrast, rats given three consecutive, subconvulsive injections of PTZ every 30 days showed a very low incidence of full kindling status (12%).

### EEG Recording

A subconvulsive PTZ treatment elicited intermittent non-ictal events that are typically dependent on the behavioral state of the animal (active or passive wakefulness)(Fig. 2D). After the second PTZ injection, the animals usually demonstrated mild multifocal body jerks (Fig. 2E). After the third PTZ injection, the animals showed typical seizure activity associated with motor arrest (Fig. 2F).

### Some of the BrdU-positive Cells are New Neurons

**Figure 3:**
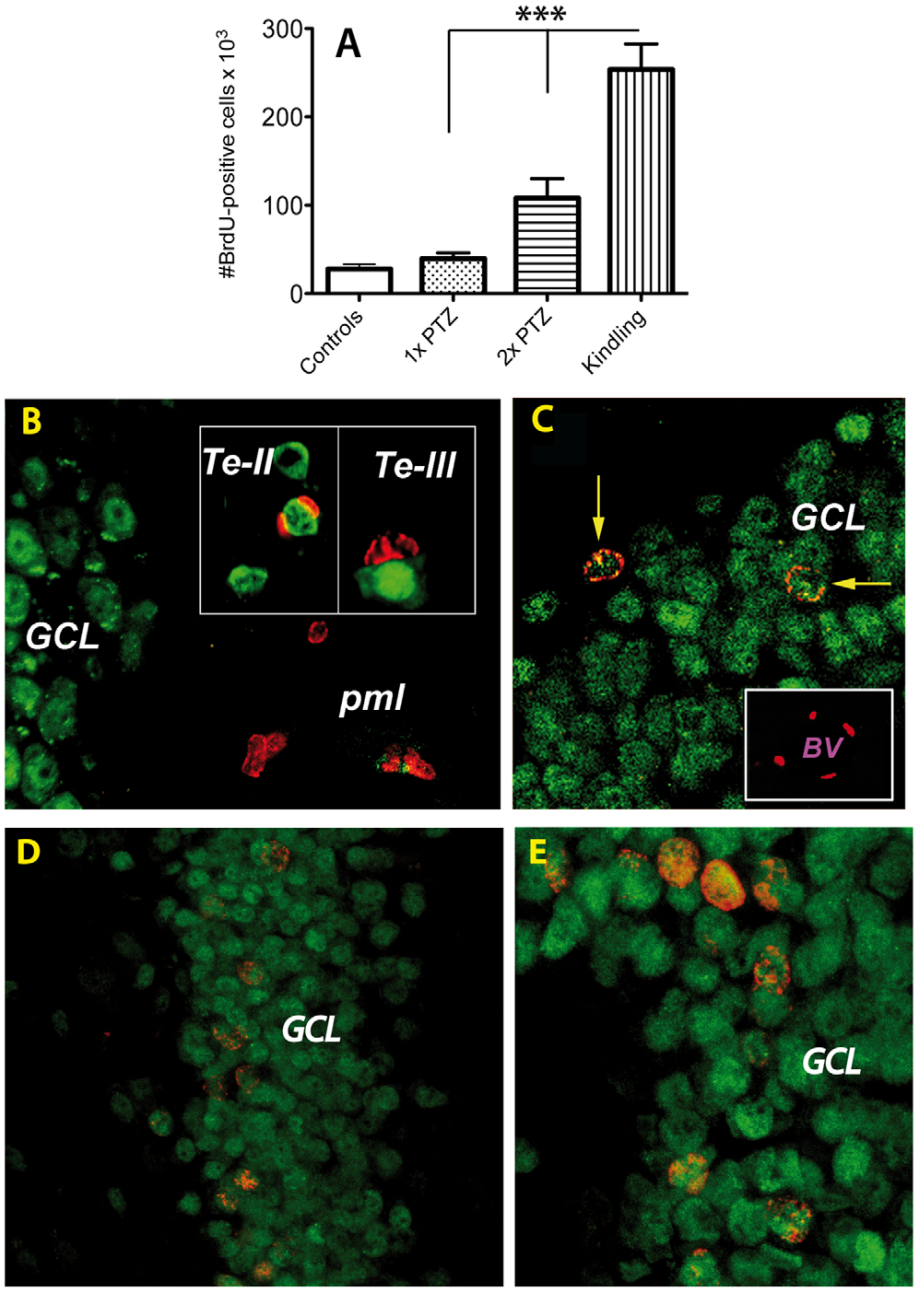
Number and phenotyping of BrdU-positive cells after seizure activity. Each PTZ treatment led to an accumulation of BrdU^+^ cells in the dentate gyrus of the kindled rats (9-fold, p = 0.0001; **A**). (**B–E**): 3D projections of confocal BrdU(red)/NeuN(green) double-labeled images from PTZ-treated animals. A single episode of seizure activity led to the appearance of BrdU-positive cells in the polymorphic layer that were in a mitosis-like state (**B**). Occasionally some neurons in layers II and III of the temporal neocortex also displayed BrdU^+^ cells in close apposition to neurons (**B**, insets). After 2× PTZ some BrdU-positive cells have differentiated into neurons, particularly in the granule cell layer (**C**, arrows). In addition, some BrdU-positive nuclei were detected in the walls of large blood vessels (**C**, inset). The number of double-labeled BrdU(red)/NeuN(green) increased with the number of PTZ injections and reached a maximum in the granule cells layer of kindled animals (**D**, low power; **E**, higher power). *Abbreviations*: *Te*, temporal neocortex; *GCL*, granule cell layer; *BV*, Blood Vessel.

Each PTZ treatment led to an accumulation of BrdU^+^ cells that were most numerous in the dentate gyrus of the kindled rats (Fig. 3A). 3D projections of confocal BrdU(red)/NeuN(green) double-labeled images from PTZ-treated animals, revealed that 50 days was sufficient time to allow some BrdU-positive cells to differentiate into neurons, particularly in the granule cell layer (Fig. 3C, arrows). The number of double-labeled BrdU(red)/NeuN(green) increased with the number of PTZ injections and reached a maximum in kindled animals (Fig. 3D, E). In the temporal neocortex, some cell nuclei also displayed a mixed NeuN-BrdU phenotype in layers II and III (Fig. 3B, insets). However, it is worth mentioning that not all BrdU-positive cells become neurons. For example, in the subgranular zone, a known neurogenic site, some of the BrdU-positive cells were incorporated into the nuclei of endothelial cells in large blood vessels (Fig.3C, inset). BrdU-positive nuclei were not found in microglia or astrocytes (not shown).

### Repeated PTZ Treatment at 25-day Intervals Led to the Accumulation of the Neuronal Lineage Marker, Doublecortin (DCX), in the Dorsal Hippocampus

**Figure 4:**
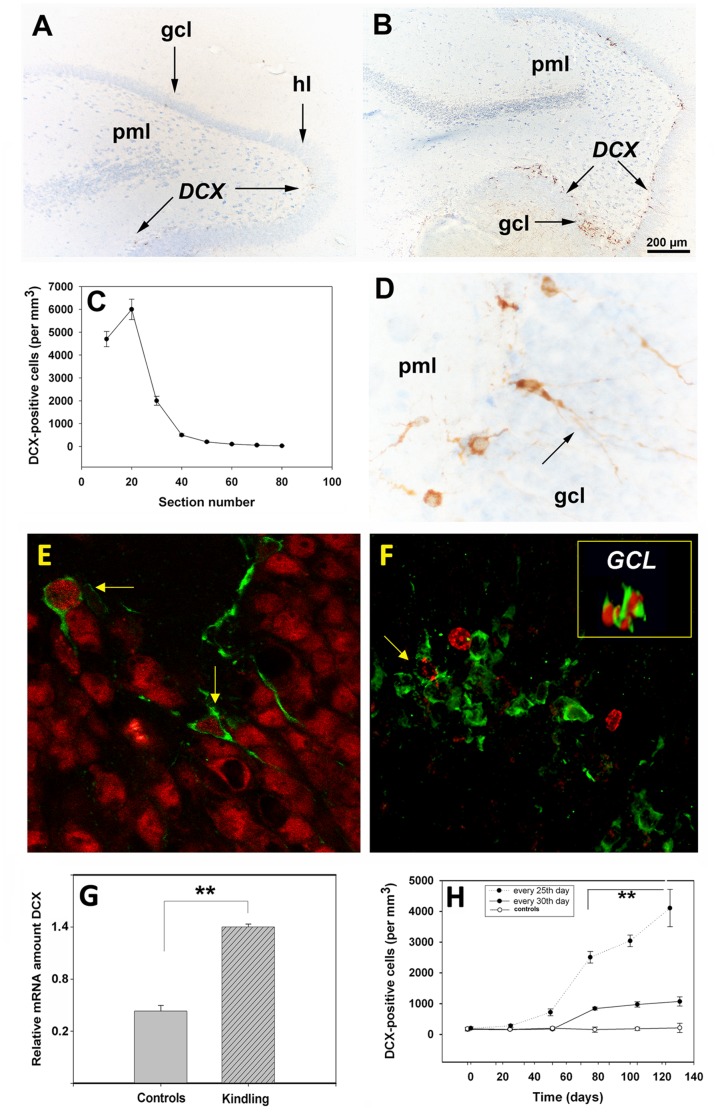
Localization and quantification of DCX in the rat brain during kindling development. (**A, B**) Overview of DCX staining in the ventral (**A,** arrows) and dorsal (**B,** arrows) hippocampal hilus of the kindled animals. The dorsal hippocampus of kindled animals, was highly significant (p = 0.001) enriched in DCX^+^-cells (**C**). Note that the DCX antigens were localized both in cell bodies and extensions penetrating the densely packed granule cell neurons (**D**, arrows). (**E-F**): Phenotyping of DCX-cells. After 2× PTZ some DCX^+^ positive cells (green) in the dorsal hippocampus along the hilar border with the granule cell layer had a NeuN nucleus (red) (**E**, arrows). In kindled animals some of the DCX (green)/BrdU (red) double-labeled cells had a clonal appearance (**F**, inset, 3D-image) while other DCX^+^ cells (green) sometimes displayed a fragmented BrdU-positivity (**F**, arrow). By quantitative RT-PCR there was a 3-fold increase (p = 0.01) in the relative amount of DCX transcripts in kindled animals over that of controls (**G**). Note that the number of DCX^+^ cells also is maximal when PTZ is administered every 25^th^ day (Fig. 4H, filled circles) as opposed to every 30^th^ day (**H**, open circles). *Abbreviations*: *gcl*, granule cell layer; *hl*, hilus; *pml*, polymorphic layer. Bars: (**A,B**), 200 µm; (**D**), 100 µm.

At day 25 following the last PTZ treatment, DCX antigens were preferentially localized at the border between the granule cell layer and hilus of the dorsal hippocampus (Fig. 4B, arrows), and only sporadically in the ventral hippocampus (Fig. 4A, arrows). Moreover, it was only the dorsal hippocampus that showed significant changes in number and phenotype of the DCX-positive (DCX^+^) cells (Fig. 4C). In enlarged images from kindled animals, it became clear that the DCX-positive cells had extensions penetrating the densely packed granule cell layer (Fig. 4D, arrows).

3D projections of DCX (green)/NeuN(red) double-labeling images from 2× PTZ treated animals indicate a clear, unexpected co-localization of DCX and NeuN-positive cells along the hilus/granule cell border in the dorsal hippocampus (Fig. 4E, arrows). 3D projections of DCX (green)/BrdU(red) double-labeling images from 1× PTZ treated animals revealed a clonal appearance of such cells (Fig. 4F, inset). With increasing time, the BrdU-labeled nucleus of DCX (green)/BrdU(red) co-labeled cells became fragmented and the number of DCX-positive cells increased dramatically in kindled animals (Fig. 4F). Intriguingly, in kindled animals the double-labeled cells also had a clonal appearance (Fig. 4F, inset).

A quantitative estimate of DCX mRNA by RT-PCR revealed, relative to control rats, a robust increase in the relative amount of DCX transcripts at day 25 after the last seizure episode in kindled animals (Fig. 4G). The actual increases are probably larger, inasmuch as dorsal-ventral asymmetry in DCX expression would have been neutralized by the homogenization of the entire hippocampus.

We previously reported that optimal kindling status is achieved when PTZ is administered within a tight time window of 25±1 day [1]. In the present study, we found that the number of DCX^+^ cells also is maximal when PTZ is administered every 25^th^ day (Fig. 4H, filled circles) as opposed to every 30^th^ day (Fig. 4H, open circles). The classical, rostral migratory stream (RMS) from the lateral ventricle to the olfactory bulb did not show significant kindling-related changes in the number of DCX-positive cells (data not shown).

### L-NAME Treatment Significantly Increases Neurogenesis and Seizure Susceptibility

**Figure 5:**
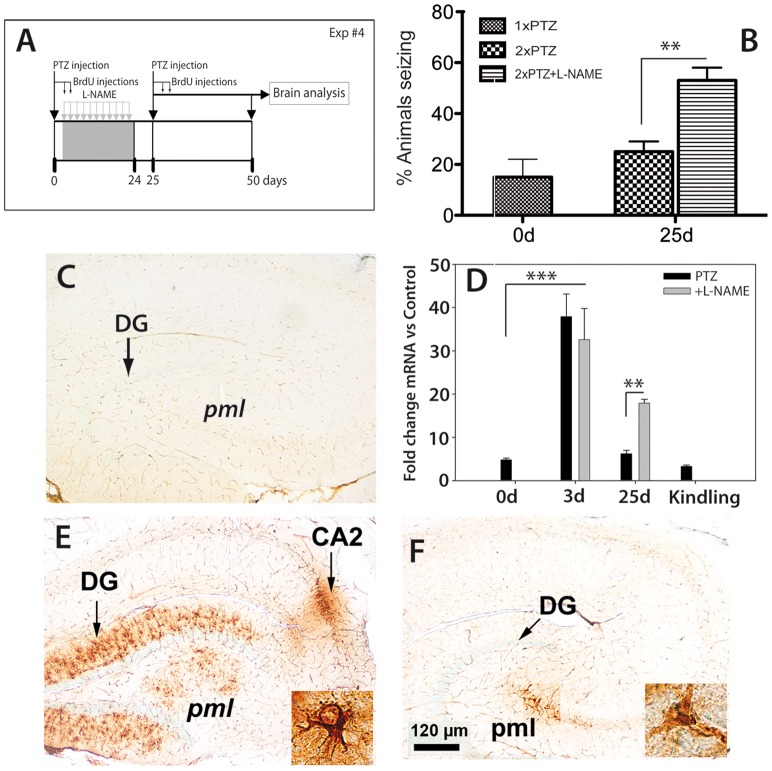
L-NAME treatment increased neurogenesis and seizure susceptibility. Daily treatment with L-NAME for 24 days (**A**) resulted in a significant increase in the number of fully seizing animals after the second PTZ administration on day 25 (**B**). In control animals, nestin immunoreactivity was detected in capillary walls (**C**). By quantitative RT-PCR there was a 7.8-fold (p = 0.001) in nestin mRNA levels at day 3 post-seizure (**D**). L-NAME-treated animals had significantly more (2.7-fold; p = 0.001) nestin mRNA than did animals treated with PTZ alone at day 25 (**D**) whereas kindled animals did not show an increased level of nestin mRNA (**D**). At the tissue level, nestin immunoreactivity at day3 in PTZ-treated animals was confined to radial glia-like cells in the inner molecular layer of the dentate gyrus, the polymorphic layer and, interestingly, to the CA2 region (arrow) (**E**, and inset). After 25 days, the nestin-like immunoreactivity was restricted to the polymorphic layer (**F** and inset). *Abbreviations*: *DG*, dentate gyrus; *pml*, polymorphic layer; *CA2*, hippocampal region.

When administered systemically, L-NAME has been shown to stimulate neurogenesis in the rat hippocampus by inhibiting NO synthase [6]–[7], [9]. We found that daily treatment with L-NAME for 24 days (Fig. 5A) resulted in a significant increase in the number of fully seizing animals after the second PTZ administration on day 25 (Fig. 5B).

An early post-seizure event was a transient increase in nestin mRNA and protein levels in the rat hippocampus. As previously described, in control animals, nestin immunoreactivity is detected in capillary walls (Fig. 5C) [8]. PTZ-evoked seizure activity led to a dramatic increase (7.8-fold) in nestin mRNA levels at day 3 post-seizure (Fig. 5D) that lasted for several days before returning to control levels by day 25. L-NAME treatment did not change PTZ-induced nestin mRNA levels at 3d post-seizure, but L-NAME-treated animals had significantly more (2.7-fold) nestin mRNA than did animals treated with PTZ alone at day 25 (Fig. 5D). Kindled animals did not show an increased level of nestin mRNA (Fig. 5D). This is not surprising, in that nestin mRNA levels were measured well after seizure activity ceased, and always shortly before a new PTZ treatment. At the tissue level, nestin immunoreactivity at day3 in PTZ-treated animals was confined to radial glia-like cells in the inner molecular layer of the dentate gyrus, the polymorphic layer and, interestingly, to the CA2 region (Fig. 5E, and inset). After 25 days, the nestin-like immunoreactivity was restricted to the polymorphic layer (Fig. 5F and inset).

### L-NAME Treatment Increased Doublecortin Levels in the Rat Hippocampus

**Figure 6:**
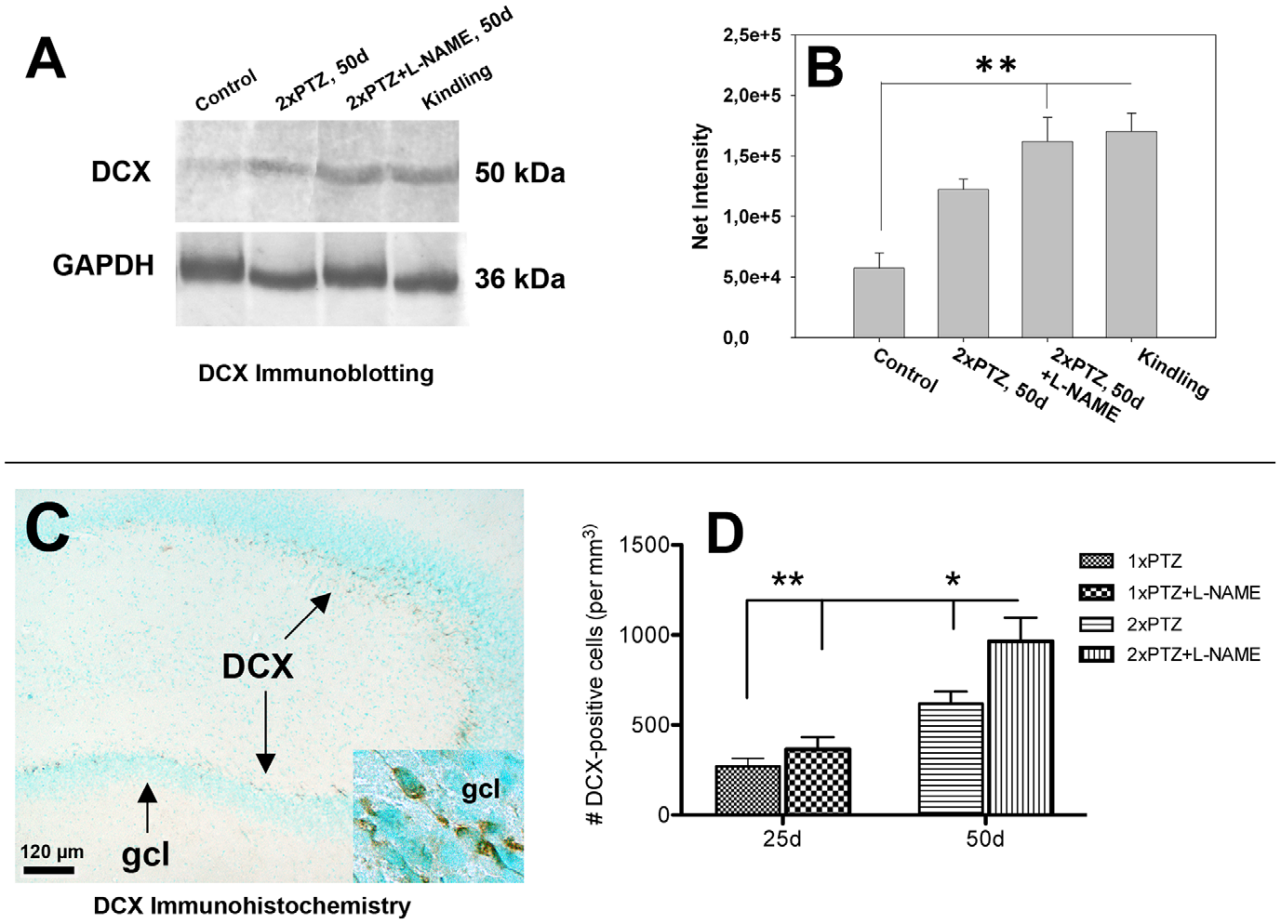
L-NAME treatment increased doublecortin levels in the rat hippocampus. L-NAME treatment increased DCX immunoreactivity on Western blots (**A**) by 2.2-fold (p = 0.02) at day 50 post-seizure (**B**). By immunohistochemistry, numerous DCX-positive cells were detected in the subgranular zone of the dorsal hippocampus by day 50 post-seizure (**C**). Quantitatively, L-NAME elicited significant increases (1.5-fold, p = 0.01) in the number of DCX-positive cells (**D**). *Abbreviations*: *gcl*, granule cell layer.

In PTZ-treated animals, DCX immunoreactivity on Western blots was increased, a phenomenon that was accentuated by the combined PTZ + L-NAME treatment at day 50 post-seizure (Fig. 6A, B). Most importantly, increased DCX levels persisted in kindled animals as well (Fig. 6A, C). Due to homogenization procedures that eliminated hippocampal asymmetry, the differences in the net intensity of DCX on western blots between control and treated animals was less evident but nevertheless significant. By immunohistochemistry, numerous DCX-positive cells were detected in the subgranular zone of the dorsal hippocampus of L-NAME-treated animals by day 50 post-seizure (Fig. 6C). Quantitatively, L-NAME elicited significant increases in the number of DCX-positive cells in the dentate gyrus by 1.5-fold (Fig. 6D).

## Discussion

In this study, we show that, at day 25±1 following stimulation of the brain by seizure-evoking pharmacological manipulations, Sprague-Dawley rats are extremely susceptible to the development of kindling status if they are subsequently primed with two subconvulsive dose of pentylenetetrazole (PTZ) given 25 days apart. Mechanistically, every 25^th^ but not 30^th^ day stimulation of the brain by PTZ enlarges the population of new, migratory neurons in the subgranular zone of the hippocampal dentate gyrus.

Because of the close resemblance to the time window of one month needed for neuronal development and maturation of newly born neurons in the adult hippocampus, we hypothesized that seizure susceptibility at day 25 is related to some critical timepoint-related integration of newly generated neurons. For this reason, we investigated systematically the time-course of cytogenesis as related to seizure susceptibility. Initially, PTZ-induced a uniform distribution of proliferating cells in many regions of the brain. The origin of these cells is not clear, but some were certainly penetrating the brain via the leptomeninges. However, the presence of a large number of BrdU-positive cells at day 3 after a single PTZ administration makes it unlikely that so many cells had been generated, or launched into a proliferative state, after seizure activity. Rather, it is reasonable to think that many of them are progenitor cells from the circulation that enter the brain via the leptomeningeal vasculature, a process facilitated by seizure activity, which can compromise the blood-brain barrier [10]. Such circulation-derived progenitor cells could become neurons [11] or even fuse with endogenous cells [12].

After 25 days, most of the BrdU-positive cells have disappeared except in the hippocampus and the temporal neocortex, suggesting a preferential survival of some BrdU-positive cells that also became DCX-positive. Upon reaching a critical threshold of cells positive for DCX (a marker of immature neurons) which was usually achieved after 3 doses of PTZ, most of the animals showed an EEG typical of seizure activity; they also achieved full kindling status, suggesting that seizure susceptibility is related both to a certain level of immature neurons and a certain developmental period. Intriguingly, we found that 2× PTZ treatment led to an increase in the number of cells in which DCX and NeuN co-localized. The presence of DCX in mature neurons also has been recently reported in the adult CNS [13].

Among early cell phenotypes, we noted the expression of the neuroepithelial and radial glia marker, nestin. Of note, nestin expression also was increased in the CA2 region of the hippocampus, suggesting input from the subiculum [14]. An early increase in proliferating radial glia-like astrocytes in the dentate gyrus after kainate-induced seizures that is consistent with a recruitment of precursors for seizure-induced neurogenesis also has been previously reported [15], [16]. However, after 24 days there were only a few nestin-like cells remaining in the polymorphic layer of the dentate gyrus. Nevertheless, at the same time, the number of DCX-positive cells had increased, a phenomenon amplified by the neurogenesis-stimulator L-NAME, suggesting that certain niches like that of the hippocampus confer selective survival capacity, and this process was reinforced after application of PTZ every 24^th^ day. Further, long-term treatment (4 wks) of rats with L-NAME has been shown to increase seizure susceptibility upon administration of a convulsant dose of pentylenetetrazole [17].

Upon reaching a certain threshold of DCX cells with deep penetrating extensions in the granule cell layer, the animals achieved kindling status. We noted that DCX is specifically expressed at 25 days along the hilar border of the granule cell layer in the *dorsal* hippocampus. Quite recently, we reported that a kindling-specific isoform of the mitochondrial Rieske protein had a similar localization in the critical time window kindling model [18]. However, it is not clear at this time how DCX expression could be related to Rieske protein expression in the dorsal hippocampus, or how Rieske protein expression is linked to neurogenesis.

The dorsal-ventral segregation of the hippocampal response to seizure has been documented in mice [19], while the dorsal-ventral hippocampal asymmetry in neurogenic response to stimulants has been recently described for rats [20] and in humans [21], [22]. Finally, some of the BrdU-positive cells became NeuN-positive and are regarded as fully mature neurons**.**

Our data are consistent with the hypothesis that seizure susceptibility is associated with the developmental status of seizure-generated new neurons in the dentate gyrus. Further, seizure susceptibility could be either related to an enhanced excitability of the newborn neurons or to a deregulation of the existing neuronal network by inappropriate connections to existing neuronal connections in the hippocampus. Consistent with the increased excitability hypothesis of newly born neurons is a recent study showing that during a maturation period of about one month, the young neurons are more excitable than the neighboring ‘older’ cells and show a lower threshold for the induction of synaptic plasticity than neighboring older granule cells [23]. Another study has shown that significant recruitment of new neurons into the existing network did not occur until they were at least 3 weeks old [4].

The enrichment of newly generated neurons by day 25 following PTZ treatment also suggests that neuronal differentiation, dendritic growth and survival of the newly born neurons in the dentate gyrus are strongly dependent on the experience of the animal during a critical period of about 3 weeks after birth [3], [24]–[27].

Earlier studies have shown that dentate granule cell neurogenesis is increased by seizures and contributes to aberrant network reorganization in the adult rat hippocampus [28]. We hypothesize that, when the integration capacity of the Sprague-Dawley rats exceeds a critical threshold, powerful new recurrent excitatory circuits can be generated in the hippocampus [29]–[31] that serve as the substrate for seizure activity. This hypothesis is supported by several recent studies that showed that dentate granule cells generated after seizure activity begin sending axons aberrantly into the dentate inner molecular layer by day 24 after stimulation [26], [32]–[33].

The involvement of PTZ in synaptic re-organization is also suggested by a recent study showing that chronic treatment with pentylenetetrazole, at non-epileptic doses, causes a recovery of cognition and long-term potentiation in Ts65Dn mice [34], [35]. Ts65Dn mice have excessive inhibition in the dentate gyrus and share several phenotypic characteristics with human Down syndrome patients.

### Conclusions

Periodic stimulation of the brain by seizure-evoking pharmacological manipulations, performed within the developmental range of hippocampal neurogenesis, enlarges the population of newly generated migratory neurons in the subgranular zone of the dentate gyrus. Such neurons can contribute to the formation of new, recurrent excitatory circuits within the hippocampal formation [36].

Our study further suggests that seizure susceptibility due to stimulus-induced neurogenesis may depend on the developmental period of post-seizure newborn neurons, and could, therefore, species-specific. For example, granule cell maturation in the dentate gyrus of a nonhuman primate (macaque monkeys) requires a minimum of a 6-mo time period, more than 6 times longer than in rodents [37]. Since development of temporal lobe epilepsy in humans may take 5–10 years to develop [38]–[41] this interval is likely to be even longer in humans. Indeed, the risk of occurrence of seizure activity in patients who have not been treated is about 50% after two years of follow-up [42].

The identification of this long, well-defined developmental interval for inducing kindling status makes possible a dissection of the cellular and genetic events underlying this phenomenon and its relationship to normal and pathological brain function.
